# The Process of Embalming

**Published:** 1873-07

**Authors:** 


### The Process of Embalming.

The Brunetti process for the preservation of the dead has recently been published in *La France Medicale*, and it consists of severaal processes:—

1. The circulatory system is cleared thoroughly out by washing with cold water till it issues quite clear from the body. This may occupy two to five hours.

2. Alcohol is injected so as to abstract as much water a possible. This occupies about a quarter of an hour.

3. Ether is then injected to abstract the fatty matters. This occupies two to ten hours.

4. A strong solution of tannin is then injected. This occupies for imbibition two to ten hours.

5. The body is then dried in a current of warm air passed over heated chloride of calcium. This may occupy two to five hours.

The body is then perfectly preserved and resists decay. The Italians exhibit specimens which are as hard as stone and perfect in form.—*Phila. Reporter*.

